# Thyroid Abscess in a Young Postpartum Female

**DOI:** 10.7759/cureus.30082

**Published:** 2022-10-08

**Authors:** Sultan Almdallaleh, Mohamed Aseafan, Latif Khan

**Affiliations:** 1 Section of Adult Nephrology, Department of Nephrology, King Saud Medical City, Riyadh, SAU; 2 Section of Medical Oncology, Department of Internal Medicine, Security Forces Hospital Program, Riyadh, SAU; 3 Department of Internal Medicine, King Saud Medical City, Riyadh, SAU

**Keywords:** thyroiditis, vancomycin, tuberculosis, thyroid, thyroid abscess

## Abstract

Due to the characteristics of the thyroid gland that prevent infiltration of pathogens, suppurative thyroid gland infections causing thyroid gland abscess are rarely encountered.

Herein, we report a young female who presented to our hospital with pulmonary tuberculosis and *Klebsiella* pneumonia complicated with empyema. During her admission, she developed a rapidly enlarging and tender thyroid gland that produced respiratory obstructive symptoms. Upon clinical examination and radiological imaging, the diagnosis of thyroid abscess was confirmed. Surgical incision and drainage of the abscess was performed along with proper coverage of antibiotics. Thereafter, the patient’s clinical status improved dramatically.

Although thyroid gland abscesses are rare in clinical practice, a rapidly enlarging thyroid gland in a patient with overt bacterial infection should raise the suspicion of thyroid abscess. Timely diagnosis and proper management can be life-saving in such cases.

## Introduction

Due to the secluded anatomical location, total encapsulation, iodine-rich environment, extensive lymphatic drainage, and abundant blood flow, thyroid gland infections causing thyroid abscess are rarely encountered [1-3]. Thyroid abscess accounts for less than 0.7% of thyroid surgical pathology [2]. Several case reports have reported that a thyroid abscess is associated with significant morbidities, including airway obstruction, sepsis, and thyroid storm. Consequently, early identification and treatments are crucial when a thyroid abscess is suspected. We present an unusual case of thyroid abscess associated with lung infection both caused by *Klebsiella pneumoniae *with confirmed pulmonary tuberculosis in a postpartum female.

## Case presentation

A 29-year-old postpartum female presented with a three-week history of undocumented intermittent fever, productive cough with odorless yellowish sputum, and shortness of breath that exacerbates with exertion of insidious onset. Her past medical history is negative for any chronic medical conditions. A month prior to her presentation, she underwent a Cesarean delivery. The patient reported a history of night sweats and significant weight loss before her pregnancy. She has a history of contact with a patient with pulmonary tuberculosis (TB) two years ago.

**Figure 1:**
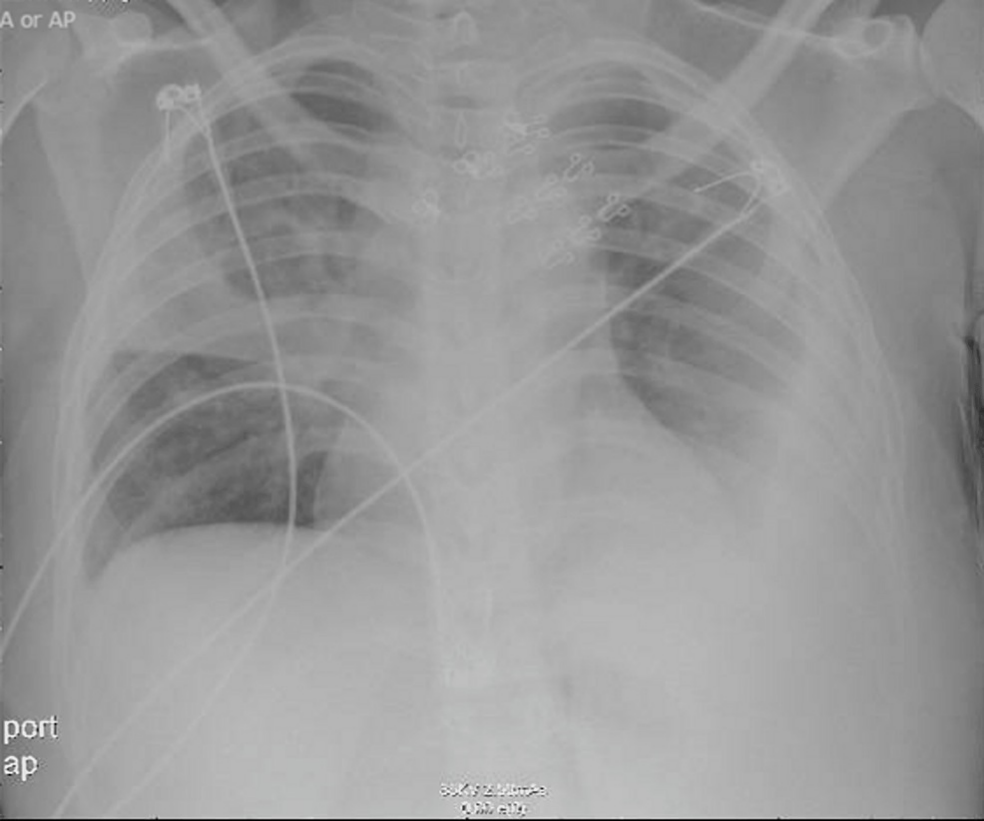
Chest radiograph on admission showing right middle lobe consolidation along with left-sided plural effusion

Her vital signs on admission showed the following: heart rate, 112 beats per minute; blood pressure, 96/60 mmHg; and oxygen saturation, 96% on room air. The physical examination signs elicited right middle zone bronchial breathing and left lower zone stony dullness on percussion with diminished breathing sounds. This was consistent with a right sided pneumonia and left pleural effusion which was later confirmed by chest X-ray. Blood investigations revealed leukocytosis (16.0 × 10^9^/L), hemoglobin of 8.9 g/dL, and erythrocyte sedimentation rate (ESR) of 120 mm/hr. Chest radiographs showed right-sided consolidation along with left-sided pleural effusion (Figure 1).

The patient was admitted as a case of pneumonia to rule out pulmonary TB. She was started on broad-spectrum antibiotics and antivirals in the form of ceftriaxone 2 gm intravenous (IV) daily and clarithromycin 500 mg by mouth (PO) every 12 hours, and oseltamivir 75 mg PO every 12 hours. A chest tube was inserted for drainge. Sputum culture grew extended spectrum beta lactamase (ESBL)-producing *Klebsiella pneumoniae*; antibiotics were switched to meropenem 1 gm IV every eight hours according to the sensitivity results. Sputum microscopy was positive for acid fast bacilli (AFB) and the pleural fluid aspirate came positive for *Mycobacterium tuberculosis* by direct polymerase chain reaction (MTB-PCR). Therefore, the patient started on an anti-TB regimen.

On day three of her admission she started to complain of anterior neck swelling that was not associated with obtructive symptoms. Neck examination revealed a palpable thyroid enlargement of both lobes associated with tenderness with no hotness or rednesss. There were no palpable cervical lymph nodes. Additionally, there were no signs or symptoms of hypo/hyperthyroidism. Moreover, the thyroid function test parameters were within normal ranges (thyroid stimulating hormone, 1.19 µL/mL; FT4, 20.71; FT3, 4.11), and antithyroglobulin antibodies were negative.

**Figure 2:**
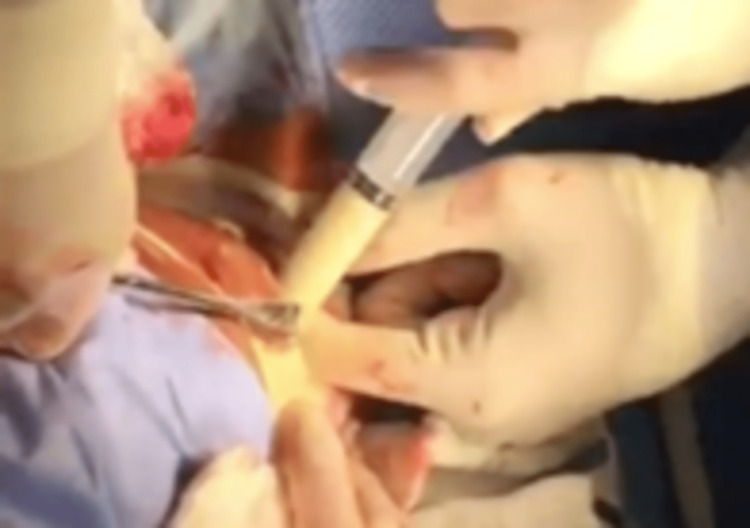
Intraoperative thyroid purulent fluid aspiration

Two days later, due to progressive enlargement of the thyroid gland, the patient experienced dysphagia to both solids and liquids, odynophagia, and difficulty in breathing. On examination, she was febrile (38.2°C), tachypneic (35 breaths per minute), and her oxygen saturation was 90% on room air. Notably, the thyroid gland was extremely tender and was more prominently enlarged on the left. Bedside needle aspiration of the thyroid gland revealed pus. A complex cystic lesion in the left thyroid lobe was confirmed by neck sonography, with several echogenic regions of heterogeneous material measuring 9 × 5 cm. The patient was immediately started on empiric vancomycin, and emergent 6 cm incision and drainage was performed under general anesthesia with delayed wound closure on day six as tertiary healing to allow complete drainage. A substantial amount of pus was aspirated, but the thyroid gland was left intact (Figure 2). *Klebsiella pneumoniae* was isolated from the aspirate, which was negative for both AFB and MTB-PCR. Patient completed 14 days of meropenem 1 gm IV every eight hours post incision and drainage. 

**Figure 3:**
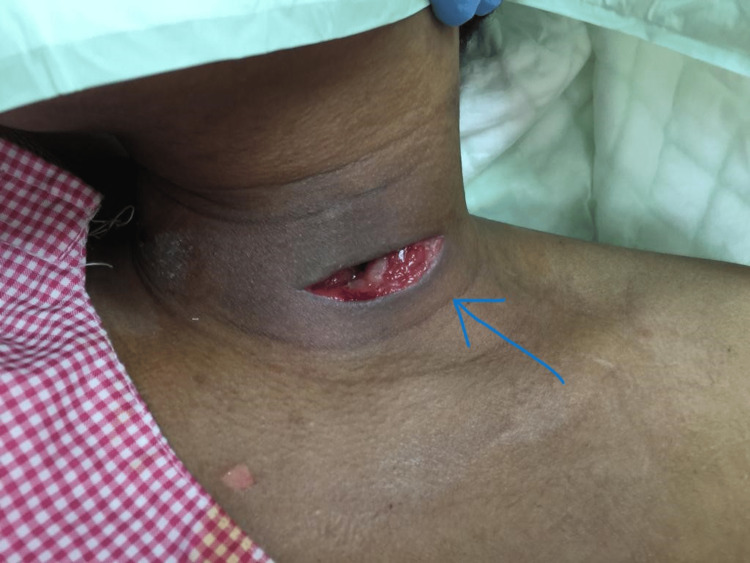
Postoperative the skin incision was left open to allow any remnant purulent discharge to drain.

Postoperatively, the obstructive symptoms were relieved, and the patient eventually recovered with no evidence of voice alteration (Figure 3). Electrolyte levels and thyroid function test parameters were all within normal limits. HIV was tested and came negative. Also immunoglobulin (IgA, IgG, and IgM) results showed normal levels. The patient was discharged home day 15 post incision and drainage on anti-TB regimen to complete the full course with follow-up in two weeks with TB clinic. 

## Discussion

Acute suppurative thyroiditis is a rare condition caused by microbial inoculation of the thyroid gland [4]. When infection progresses, an abscess may form within the thyroid tissue [5].

The most common organisms that cause thyroid abscess are *Staphylococcus aureus* and *Streptococcus pneumonia* [6]. Less commonly encountered organisms include *Klebsiella *spp. [7], *Salmonella *spp. [8], and *Acinetobacter* [9]. Therefore, empiric antibiotics should cover for gram-positive organisms until the culture and sensitivity reports are available [5].

Although extremely rare, cases of tuberculous thyroid abscesses have been reported in the literature [10]. In our case, although our patient’s primary impression was pulmonary TB, there was no evidence of tuberculosis in the thyroid aspirate.

The main pathogenic mechanism of this disease seems to be hematogenous dissemination from a focus of infection in the oropharynx or respiratory tract to a thyroid gland with a pre-existing abnormality [11]. In our case, since the same organism was isolated from both the sputum and abscess, we hypothesized that infection disseminated hematogenously from the respiratory tract. However, there was no evidence of thyroid gland pathology.

The diagnosis of a thyroid abscess is usually delayed because the presenting features, including fever, neck pain, and odynophagia, are nonspecific and mimic other clinical entities such as acute pharyngitis [5,11]. Other than these symptoms, our case was also complicated with airway obstruction as evidenced by dyspnea and desaturation.

Neck sonography and computed tomography are both sensitive in diagnosing a thyroid abscess [6,12]. In our case, bedside needle aspiration and neck sonography confirmed the diagnosis of a thyroid abscess.

## Conclusions

Although a thyroid abscess is rare, especially in immunocompetent patients, in clinical practice, a rapidly enlarging thyroid gland should raise the suspicion of a thyroid abscess. Prompt diagnosis and early surgical intervention along with proper microbial coverage of antibiotics is essential in such cases. As a thyroid abscess is a complex condition, it may require a multidisciplinary approach and involve internal medicine, endocrinology, general surgery, and radiology services.
